# Atomistic Insights into the Reactive Diffusion of CO_2_ in Guanidine-Based Facilitated Transport Membranes

**DOI:** 10.1021/acs.jpcc.5c01717

**Published:** 2025-05-11

**Authors:** Changlong Zou, Xuepeng Deng, Yang Han, Li-Chiang Lin

**Affiliations:** a William G. Lowrie Department of Chemical and Biomolecular Engineering, 2647 The Ohio State University, 151 West Woodruff Avenue, Columbus, Ohio 43210-1350, United States; b Department of Chemical Engineering, 33561 National Taiwan University, No.1, Sec. 4 Roosevelt Rd., Taipei 106319, Taiwan

## Abstract

The pressing need to address climate change has led to significant advancements in carbon dioxide (CO_2_) capture technologies. Notably, facilitated transport membranes (FTMs) are distinguished by their exceptional selectivity and permeance, attributed to their reversible chemical reactions with CO_2_. This study, for the first time, sheds light on the reactive diffusion mechanism of CO_2_ in FTMs, utilizing 1,1,3,3-tetramethylguanidine (TMG) as a mobile carrier. Specifically, state-of-the-art molecular dynamics (MD) simulations, augmented by a reparameterized reactive force field (ReaxFF) capable of describing atomistic interactions and reaction pathways, are conducted to investigate the transport of CO_2_ in TMG. The analysis of mean squared displacement (MSD) and diffusion coefficients reveals a clear hierarchy in the mobility of reaction components. Our findings highlight a unique hopping diffusion mechanism between bicarbonate ions and TMG molecules, increasing the diffusivity of reacted CO_2_ by 1.4 times. The hopping events observed not only enhance our understanding of molecular mobility but also offer a means to boost the performance of FTMs in CO_2_ capture applications. Overall, this research lays the groundwork for the future design of FTMs with optimal carrier properties.

## Graphical abstract

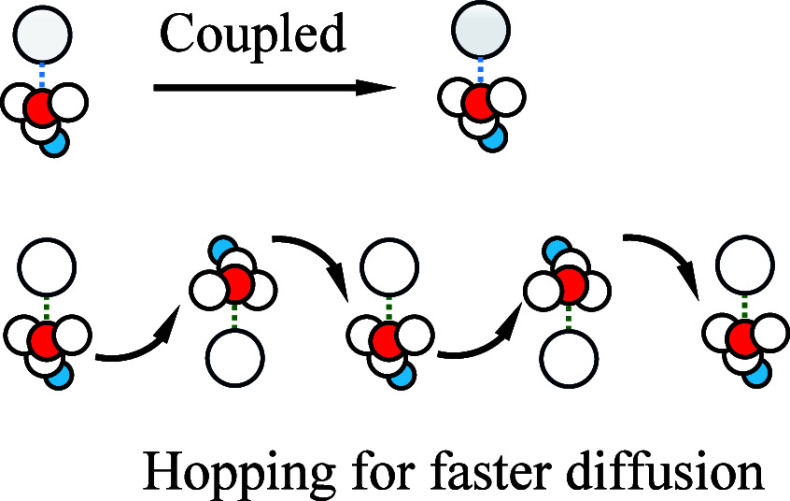

## Introduction

Polymeric membrane-based CO_2_ separation processes, particularly with facilitated transport membranes (FTMs), have emerged as a promising avenue for their substantially enhanced permeance of CO_2_ and selectivity over N_2_ via introducing reversible chemical reactions between the membranes’ reactive functional groups and CO_2_.^1–12^ In most FTMs, the separation performance is largely governed by mobile carriers—small molecular amines dispersed in a polymer matrix.^7,10,12–15^ When these mobile carriers prioritize the formation of bicarbonate instead of carbamate, the equimolar stoichiometry of the former enhances CO_2_ sorption and consequently improves both permeance and selectivity.^12,14,16,17^ As a result, there has been considerable interest in developing novel mobile carriers to optimize chemisorption of CO_2_. Aside from enhancing the sorption capability, it can be intuitively anticipated that systems with a higher mobility of CO_2_-related species may also demonstrate a better performance. Understanding their diffusion behaviors and elucidating the reactive diffusion mechanism at the atomic scale are therefore crucial. However, studies on this fundamental aspect remain largely limited.

In amine-containing FTMs, CO_2_ can exist and diffuse in multiple forms. They include unreacted molecule, mobile carrier–CO_2_ complex (i.e., zwitterion and carbamate), and bicarbonate.^2,14,18^ Inherently, the dynamic interconversion among these diffusing species makes the deconvolution of the reactive diffusion extremely challenging. Unreacted CO_2_ molecules are expected to follow the solution-diffusion mechanism.^2,9,10,12,16,19,20^ When CO_2_ molecules react with mobile carriers (i.e., referred to as reacted CO_2_ hereafter) following the carbamate pathway, two mobile carriers are involved in reacting with one CO_2_, yielding a large carbamate ion and a corresponding protonated carrier (i.e., an ammonium ion). This pathway is typically favored when sterically unhindered amines are used as carriers, particularly under low-water or dry conditions.^14,21–24^ The mobility of the reacted CO_2_ relies on either the coupled diffusion of the ion pair or the cleavage of the carbamate bond (Figure 1(a)).^16,17,19–23^ It is reasonable to infer that the latter events are associated with a high activation energy and thus less likely. By contrast, the bicarbonate pathway becomes dominant in the presence of sterically hindered amines or other stronger bases in the presence of sufficient amount of water.^14,18,25^ Following the bicarbonate pathway, only one mobile carrier reacts with a single CO_2_ molecule, producing a small anion and a protonated carrier. Similar to carbamate, bicarbonate can form electronic associations with the protonated mobile carrier and diffuse in tandem. However, owing to its significantly smaller size, bicarbonate may also exhibit a “hopping” motion, wherein it transfers between adjacent protonated mobile carriers (Figure 1(b)). These hypothesized hopping events are anticipated to enhance its mobility across the membrane, as they allow bicarbonate to not only diffuse alongside a given mobile carrier but also jump between neighboring ones. As experimentally observed and noted above, FTMs with carriers favoring bicarbonate formation generally exhibit higher CO_2_ permeance than those favoring carbamate formation.^18,26^ While this enhancement is typically attributed to the greater amine efficiency of the bicarbonate pathway, the molecular perspective on the bicarbonate diffusion process might offer an alternative explanation for or contribute in part to this observed advantage. Understanding these mechanisms will also provide valuable insights for advancing the design of mobile carriers in future applications.

**Figure 1:**
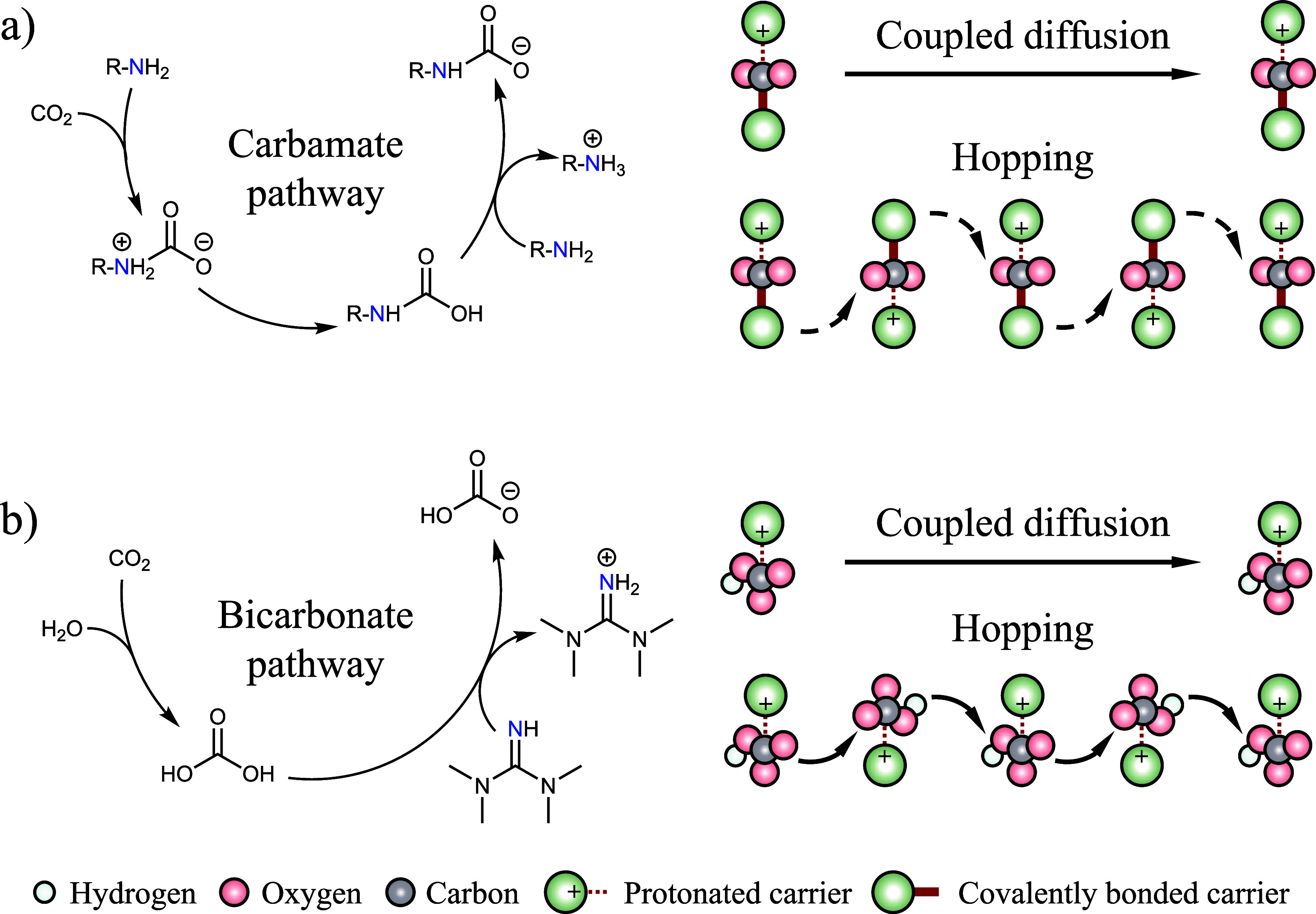
Schematic illustrations of reactive diffusion mechanisms for CO_2_ with mobile carriers in the forms of (a) carbamate (i.e., the coupled diffusion and the unlikely hopping mechanism involving the cleavage of the carbamate bond) and (b) bicarbonate (i.e., both the coupled diffusion and the hopping mechanisms) in an FTM.

To explore the diffusion of CO_2_-related species at an atomic level, this study leverages state-of-the-art computational approaches. Techniques, such as density functional theory (DFT) and classical molecular dynamics (MD), have shown the capability to offer molecular understandings of various properties such as reaction chemistry, diffusivity, and gas solubility.^21^ However, these approaches face challenges in addressing the reactive diffusion of CO_2_ within the membrane. Employing the former to study a complex system is simply prohibitive for its tremendous computational cost, while the latter lacks the capability to simulate the chemical reactions essential to modeling the system of interest. In this context, MD employing a bond-order-based reactive force field (ReaxFF), which was first developed by Goddard and co-workers,^27^ enables the explicit description of bond association and dissociation in large systems. To this end, this work conducts, for the first time, ReaxFF MD simulations to elucidate the reactive diffusion of bicarbonate in FTMs. As noted above, since both carbamate- and bicarbonate-mediated transport can occur in membranes containing amine carriers,^9,22,24,25^ isolating and analyzing CO_2_ diffusion following the bicarbonate pathway alone presents a significant challenge. To overcome this, we herein study 1,1,3,3-tetramethylguanidine (TMG), which strongly favors the bicarbonate pathway,^28,29^ as the mobile carrier. To ensure accurate description of the interactions between CO_2_ and TMG as well as their reaction, an optimized set of ReaxFF parameters is also developed. With the optimized ReaxFF, the mean squared displacement (MSD) of molecules collected from MD simulations reveals a clear hierarchy in the mobility of different components and more importantly, confirms the unique hopping diffusion mechanism between bicarbonate ions and TMG molecules. This work not only showcases a method for directly observing reactive diffusion pathways but also deepens our fundamental understanding of CO_2_ diffusion in FTMs, aiding the future design and selection of mobile carriers.

## Computational Methods

This section describes computational methods employed in this study, offering details of MD simulations, ReaxFF validation and reparameterization, DFT calculations, determinations of MSD, and diffusivity calculations. It should be noted that the analyses presented herein explicitly exclude the polymer matrix, as mobile carriers typically constitute the predominant fraction of all carriers in FTMs (i.e., as large as 85 wt %).^25,26,30^ Our previous work has demonstrated that omitting the polymer matrix does not distort the sorption and diffusion characteristics of CO_2_ within the membrane.^14^ Additionally, the absence of polymer enables more direct observation of reaction-diffusion coupling and hopping behavior without confounding effects. While this simplification limits the direct transferability of the findings to highly cross-linked or rigid polymer environments, we believe the mechanistic insights obtained here are broadly applicable to a wide range of hydrated FTM systems where mobile carriers govern transport.

### MD Simulations

Both classical and ReaxFF MD simulations are conducted using the open-source LAMMPS^31^ package. Three systems, including bulk phase TMG, a single TMG molecule, and CO_2_/TMG/H_2_O mixtures, are studied. The bulk phase TMG system is used to assess the intermolecular interactions between TMG molecules. The system is constructed with 200 TMG molecules in a periodic box with dimensions of 30 Å × 30 Å × 30 Å, followed by a proper relaxation as will be detailed later. The single TMG system is used to assess the intramolecular interaction of a single TMG molecule, such as its bond stretching and angle bending. It is constructed with a single TMG molecule in a box with dimensions of 24 Å × 24 Å × 24 Å. The CO_2_/TMG/H_2_O ternary systems are also investigated to probe the reactive diffusion of CO_2_ within TMG in the presence of water. These systems comprise 200 TMG molecules, 1300 water molecules, and varying numbers of CO_2_ (i.e., 10, 25, 45, 55, and 70 CO_2_ molecules) to simulate the systems at a water loading of 50 wt % with varying CO_2_ concentrations (i.e., 1.0, 2.5, 4, 5, and 6 wt %, respectively).

In these calculations, the simulation domain is first equilibrated using classical force fields. Both nonbonded and bonded contributions are included to describe intermolecular and intramolecular interactions. For the former, the 12–6 Lennard-Jones (L-J) potential for van der Waals interactions and static point charge models for long-range Coulombic interactions are used. The generalized Amber force field (GAFF)^32^ is employed to model TMG, while water molecules are represented by the extended simple point charge (SPC/E) model.^33^ CO_2_ is described with the transferable potentials for phase equilibria (TraPPE) model.^34^ The L-J potential is truncated and shifted to zero at a cutoff radius of 12 Å, while the long-range electrostatic interactions are computed using the particle–particle particle-mesh (pppm) method with a precision of 10^–6^. The geometric mixing rule is applied to estimate the pairwise L-J parameters. For bonded interactions, harmonic models are adopted for bonding and bending with the Fourier style for dihedral contributions. The 1–4 intra-vdW interactions are also included with a scaling factor of 0.5. Simulations in the canonical ensemble are conducted at 330 K with the temperature modulated by the Nosé-Hoover thermostat with a damping factor of 100 time steps (i.e., 100 fs) for at least 10 ns.

ReaxFF MD simulations are then conducted to study the physical and chemical properties of the above-mentioned systems. Unlike classical force fields, ReaxFF is a bond-order-dependent potential and is capable of describing bond association and dissociation, as shown in Equation 1.

$$U=E_{bond}+E_{over}+E_{under}+E_{val}+E_{pen}+E_{tors}+E_{conj}+E_{vdW}+E_{coulomb}$$ 1

The bond energy (*E*_*bond*_) can be calculated in terms of bond order *BO*_*ij*_ (Equation 2), which is expressed in terms of the interatomic distance *r*_*ij*_ as well as correction terms *f* for overcoordination and residual 1–3 bond orders in valence angles (Equations 3 and 4).^27^

$$E_{bond}=-D_{e}\cdot BO_{ij}\cdot e^{p_{be,1}\left(1-BO_{ij}^{p_{be,1}}\right)}$$ 2

$$BO_{ij}=BO_{ij}^{\prime }\cdot f_{1}\left(\Delta_{i}^{\prime },\Delta_{j}^{\prime }\right)\cdot f_{4}\left(\Delta_{i}^{\prime },BO_{ij}^{\prime }\right)\cdot f_{5}\left(\Delta_{j}^{\prime },BO_{ij}^{\prime }\right)$$ 3

$$BO_{ij}^{\prime }=e^{\left(p_{bo,1}\cdot {\left(\frac{r_{ij}}{r_{o}}\right)}^{p_{bo,2}}\right)}+e^{\left(p_{bo,3}\cdot {\left(\frac{r_{ij}^{\pi }}{r_{o}}\right)}^{p_{bo,4}}\right)}+e^{\left(p_{bo,5}\cdot {\left(\frac{r_{ij}^{\pi \pi }}{r_{o}}\right)}^{p_{bo,6}}\right)}$$ 4

In these equations, *D*_*e*_ and *p* are fitted parameters. The correction terms *f* depend on the bond orders and deviation degrees of the sum of the uncorrected bond orders around an atomic center from its valency.^24^ *E*_*over*_ and *E*_*under*_ are energy correction terms that respectively handle the excess degrees of over and under coordination remaining in the molecule after the correction of bond order. *E*_*val*_ and *E*_*pen*_ are the energy terms contributed by valency angle energy and penalty energy from the system with two double bonds sharing an atom, respectively. *E*_*tors*_ *, E*_*conj*_ *, E*_*vdW*_ *,* and *E*_*coulomb*_ are respectively energy contributions from angle torsion, conjugation effects, nonbonded van der Waals interactions, and Coulomb interactions.^24^

### Force Field Validation

To evaluate the accuracy of ReaxFFs, several computed quantities, including bulk phase density, intramolecular interactions, and reaction properties with CO_2_, are benchmarked against experimental data or results from DFT calculations. The validation begins by evaluating the ability of an adopted ReaxFF to reproduce the intermolecular interactions of TMG in its bulk phase, characterized by its density of 0.92 g/cm^3^.^35^ Three independent MD simulations are conducted for the bulk phase TMG system, described by a given employed ReaxFF, in the NPT ensemble with each lasting for at least 10 ns. The density is computed from the last 2 ns of each simulation, followed by averaging results from the three calculations.

To assess the capability of the ReaxFFs in describing the intramolecular interactions of a single TMG molecule, a set of 4000 configurations is employed with their reference energies determined by DFT implemented in Gaussian 16 ^36^ using a combination of the B3LYP (Becke three-parameter Lee–Yang–Parr)^37^ functional and the 6–311++G­(d,p) basis set. Although DFT methods inherently carry systematic errors, this combination has been widely adopted in studies of CO_2_–amine reactions for its reliable qualitative prediction of reaction energetics.^14,18,25,38–40^ These configurations are derived from classical MD simulations with the GAFF force field^32^ in a canonical ensemble at two different temperatures (i.e., 330 and 500 K, each with 2000 configurations). Besides, to specifically evaluate the performance of the ReaxFFs on specific bonds or bends, a series of testing configurations are also generated by adjusting their bond lengths or bend angles away from their equilibrium values while maintaining the rest of the molecular structure unchanged. Their DFT-computed and ReaxFF-determined energies are then compared.

Aside from these physical-related properties, the accuracy of the adopted ReaxFF in describing the reaction pathway between CO_2_ and TMG is also probed. As known, a CO_2_ molecule can react with the conjugated nitrogen on TMG following either the carbamate pathway or the bicarbonate pathway^41^ as shown in Equations 5–6 and Equations 7, respectively.

$${\mathrm{CO}}_{2}+(N({\mathrm{CH}}_{3}{)}_{2}{)}_{2}\mathrm{C=NH}⇌(N({\mathrm{CH}}_{3}{)}_{2}{)}_{2}C{\mathrm{=NH}}^{+}{\mathrm{-COO}}^{-}$$ 5

$$(N({\mathrm{CH}}_{3}{)}_{2}{)}_{2}\mathrm{C=}{\mathrm{NH}}^{+}{\mathrm{-COO}}^{-}+(N({\mathrm{CH}}_{3}{)}_{2}{)}_{2}\mathrm{C=NH}⇌(N({\mathrm{CH}}_{3}{)}_{2}{)}_{2}\mathrm{C=N}{\mathrm{-COO}}^{-}+(N({\mathrm{CH}}_{3}{)}_{2}{)}_{2}C{{\mathrm{=NH}}_{2}}^{+}$$ 6

$${\mathrm{CO}}_{2}+H_{2}O+(N({\mathrm{CH}}_{3}{)}_{2}{)}_{2}\mathrm{C=NH}⇌(N({\mathrm{CH}}_{3}{)}_{2}{)}_{2}C{{\mathrm{=NH}}_{2}}^{+}+{{\mathrm{HCO}}_{3}}^{-}$$ 7

DFT calculations are again employed to compute the reference energy of reactants, intermediate complexes, transition-state (TS) structures, and products along each reaction pathway. In these calculations, reactants are assumed to be independent molecules, while complexes, TS structures, and products are treated as combinations of molecules positioned in energetically favorable arrangements. Geometry optimization and frequency analysis are performed for each structure; the structures of reactants, complexes, and products are optimized until no imaginary (i.e., negative) frequency can be found, while the TS structures are optimized until only one imaginary frequency remains. It should be noted that water is essential in these reactions; aside from directly participating as a reactant in the bicarbonate pathway, it also stabilizes both the intermediates and products. In this study, a hybrid approach of incorporating both implicit and explicit water solvation models is employed. The implicit water solvation is modeled by the IEFPCM (integral equation formalism polarizable continuum model),^42^ a model that has been adopted in previous studies on the CO_2_–amine reactions, such as those by Narimani et al.^43^ and Davran-Candan.^41^ The explicit water solvation model uses sufficient water molecules surrounding the reactive molecules. As detailed in Supporting Information (SI), the hybrid implicit-explicit water solvation model with eight explicit water molecules offers a good balance in accuracy and efficiency.

### ReaxFF Reparameterization

ReaxFFs are generally highly system-specific, and none of the existing ReaxFFs can reproduce the aforementioned physical and chemical properties, as will be detailed in a later section. Therefore, reparameterization of ReaxFF is necessary. With the data set employed for ReaxFF validation at our disposal, Figure 2 depicts the reparameterization procedure. This process involves several major steps that iteratively improve the accuracy of the force field. Specifically, the intermolecular training is first conducted by adjusting the van der Waals parameters associated with carbon (C) and nitrogen (N) atoms until the bulk phase TMG density predicted by the refined ReaxFF deviates by no more than 0.1 g/cm^3^ from the experimental density. Subsequently, GARFfield,^44^ a genetic algorithm-based reactive force field optimizer method, is employed to optimize the force field parameters related to C, N, and hydrogen (H) atoms, as well as all bonds, angles, and dihedrals presented in TMG until achieving a mean absolute error (MAE) of less than 5 kcal/mol for the intramolecular training set of 4000 configurations. Once achieved, a new intramolecular test set, consisting of another 4000 configurations, is generated using the newly optimized ReaxFF. The DFT-computed and ReaxFF-determined potential energies for each configuration in this new set are then compared. If the MAE exceeds 5 kcal/mol, another round of optimization will be conducted. This process will be iteratively conducted until the MAE is lower than 5 kcal/mol. The reparameterization process then proceeds to ensure that the CO_2_–TMG reaction pathways can be appropriately captured. Particularly, parameters associated with oxygen (O) atoms are optimized until the MAE for the energies along the reaction pathways is also less than 5 kcal/mol. The obtained ReaxFF will then be used to generate another intramolecular testing set to verify if the molecular configurations of TMG can still be adequately described. If not, another round of intramolecular training must be conducted. These optimizations are performed iteratively until the MAE values for both the intramolecular set and the reaction energies are less than 5 kcal/mol.

**Figure 2:**
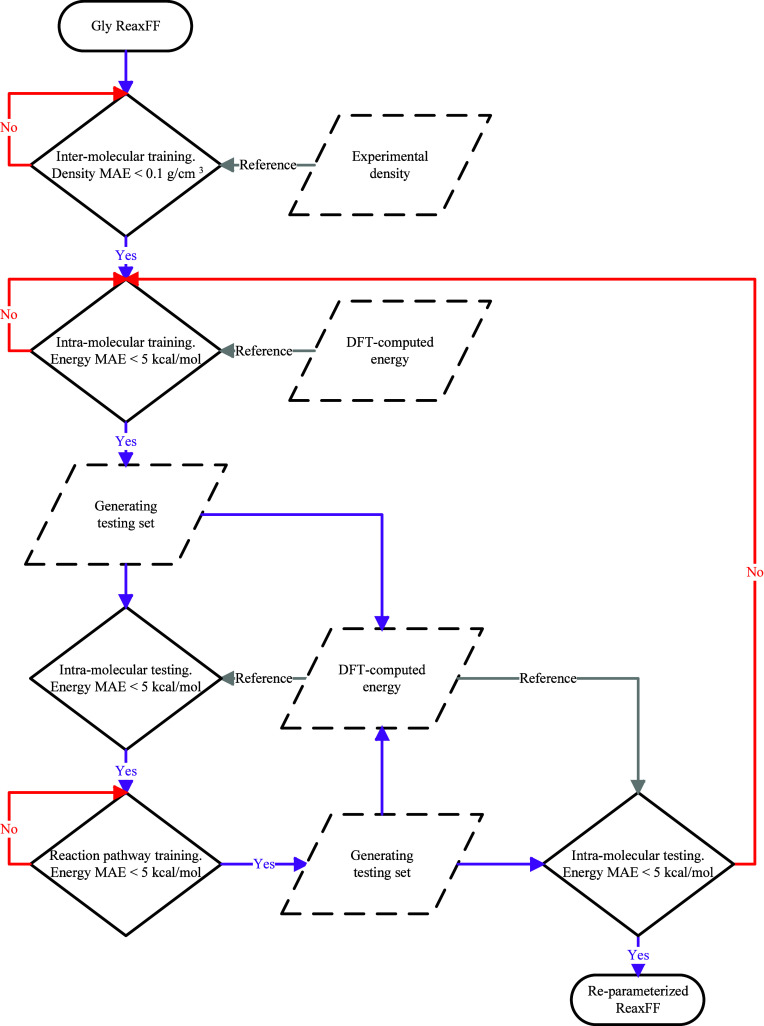
Flowchart of ReaxFF reparameterization.

### Calculation of MSD and Diffusivity

MD simulations with the reparameterized ReaxFF are conducted in a NVT ensemble for at least 5 ns at 330 K. The collected trajectories are analyzed to calculate the MSD with an in-house script for each component involved in the system. Their respective self-diffusivities are subsequently determined based on Einstein’s relation as shown in Equation 8:

$$MSD=\frac{1}{N}\sum_{j=1}^{N}{\left|r_{i,j}\left(t\right)-r_{i,j}\left(0\right)\right|}^{2}=6D_{i}t$$ 8

where *D*_*i*_ is the self-diffusivity of the species *i*, *N* is the its total number of molecules, *r*_*i,j*_ *(t)* represents the position of the center of mass for its j^th^ molecule (i.e., *j* from 1 to N) at time *t*. The diffusion region is established when the slope of ln­(MSD) vs ln­(*t*) lies between 0.95 and 1.05. Specifically, the one having a slope closest to 1 is used to calculate the *D*_*i*_. Furthermore, the local environment of each CO_2_ molecule is characterized by identifying and tracking nearby TMG molecules within 8 Å, defined as those where the distance between the C atom of CO_2_ and the conjugated N in TMG is less than 8 Å.

## Results and Discussion

In this section, the accuracy of currently available ReaxFFs in modeling the systems of interest is first discussed, followed by introducing a newly reparameterized ReaxFF. Subsequently, the diffusion behavior of CO_2_ within a bulk TMG phase in the presence of water is analyzed, with a particular focus on the mobility and diffusion of reacted CO_2_ molecules.

### Performance of Currently Available ReaxFFs

While ReaxFFs are highly effective in describing systems involving reactions, they are inherently system-specific and often require reparameterization to model different or even closely related systems. To date, force fields including the glycine ReaxFF^45^ (i.e., denoted as the Gly ReaxFF hereafter), the tetrabutylphosphonium glycinate ReaxFF^46^ (i.e., denoted as the TGly ReaxFF), the hydrocarbon/water ReaxFF^47^ (i.e., donated as the Hydro ReaxFF), and the biodegradation ReaxFF^48^ (i.e., donated as the Bio ReaxFF) have been reported in the literature. These ReaxFFs contain framework on C, H, N, and O elements, which are required to describe the CO_2_/TMG/H_2_O systems. While they were developed to describe molecules with structural similarities to TMG, Table 1 demonstrates that none can reproduce both the bulk phase density (i.e., 0.92 g/cm^3^)^35^ and the intramolecular interaction of a single TMG molecule.

**Table 1:** Bulk TMG Densities Computed Using Existing ReaxFFs, along with the MAE of Their Predicted Intra-molecular Interactions in a Single TMG Molecule Relative to DFT Calculations

ReaxFF	MD-computed bulk phase density (g/cm^3^)	MAE of intramolecular interactions (kcal/mol)
Gly	1.36	17.5
TGly	0.97	25.8
Hydro	0.98	27.7
Bio	1.18	30.8

Table 1 shows that both the Gly ReaxFF and the Bio ReaxFF predict a substantially higher bulk phase density than the experimental reference, while the TGly ReaxFF and the Hydro ReaxFF appear to perform reasonably well. Though, all of them fail in describing the intramolecular interactions of a TMG molecule; the Gly ReaxFF has a large MAE of 17.5 kcal/mol, while TGly, Hydro, and Bio ReaxFFs show even higher MAE values of 25.8, 27.7, and 30.8 kcal/mol, respectively. The significant deviation of the intramolecular interaction calculated by the Gly ReaxFF as compared to that determined by DFT can be further visualized in Figure 3. Besides, it is also found that these ReaxFFs cannot describe the bond and angle energies of TMG, as shown in Figure 4 and Figure S2. While most ReaxFF models adequately describe the N–H bond, they fail in the N=C_c_ bond as well as both the N–C_c_–N and H–C_c_=N angles (see atom labels in Figure S1). Other bonds and angles depicted in Figure S2 also show varying levels of deviations. Moreover, even the best-performing model, the Gly ReaxFF, fails to accurately capture the CO_2_–TMG reaction pathways, as will be discussed in greater detail later. Collectively, these results highlight the necessity for a reparameterized ReaxFF. Given that the TGly ReaxFF, Hydro ReaxFF, and Bio ReaxFF were all derived from the Gly ReaxFF—and that the Gly ReaxFF demonstrates relatively better performance—parameters of the Gly ReaxFF are employed as the starting point for the force field reparameterization.

**Figure 3:**
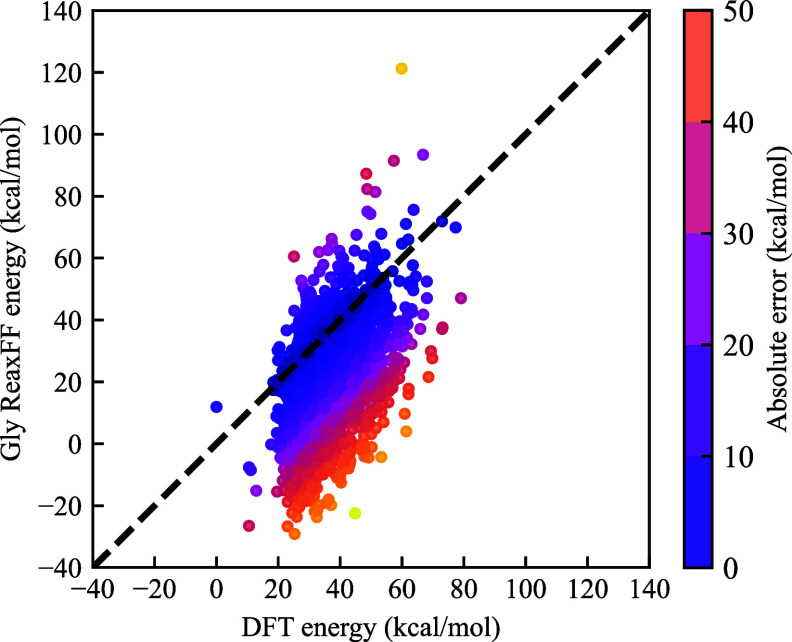
Comparison of the intramolecular interactions computed by the Gly ReaxFF and DFT for 4000 configurations of a single TMG molecule.

**Figure 4:**
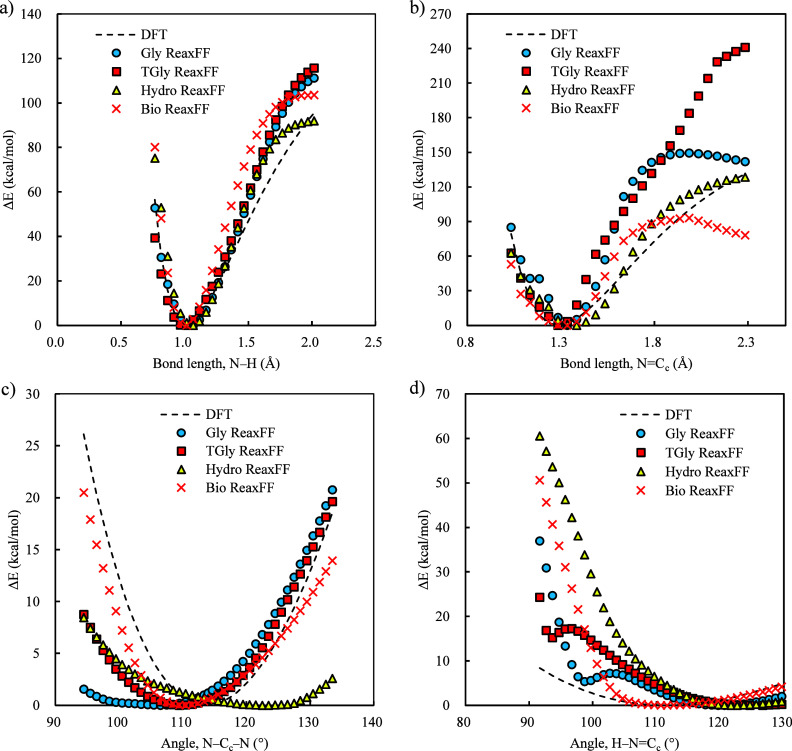
Comparison of bond ((a) N–H and (b) N=C_c_) and angle ((c) N–C_c_–N and (d) H–N=C_c_) energies calculated by DFT and those by existing ReaxFFs. The center C atom of TMG is donated as C_c_, with additional atom label definitions shown in Figure S1.

### Newly Reparameterized ReaxFF

With the procedure shown in Figure 2, a reparameterized ReaxFF, denoted as the TMG ReaxFF with parameters provided in the SI, is obtained. This force field is capable of predicting the bulk phase density of TMG (i.e., 0.96 g/cm^3^; c.f., experimental reference: 0.93 g/cm^3^), describing intramolecular interactions with an MAE of 2.9 kcal/mol, and reproducing the energy profiles along reaction pathways with an MAE of 4.6 kcal/mol. As can be seen from Figure 5(a), the intramolecular interactions calculated by the TMG ReaxFF resemble those computed by DFT and are notably more accurate than the Gly ReaxFF (i.e., MAE of 17.5 kcal/mol). The optimized TMG ReaxFF can also reproduce bond and angle energies, as shown in Figure 5 **(b–c)** and Figure S3.

**Figure 5:**
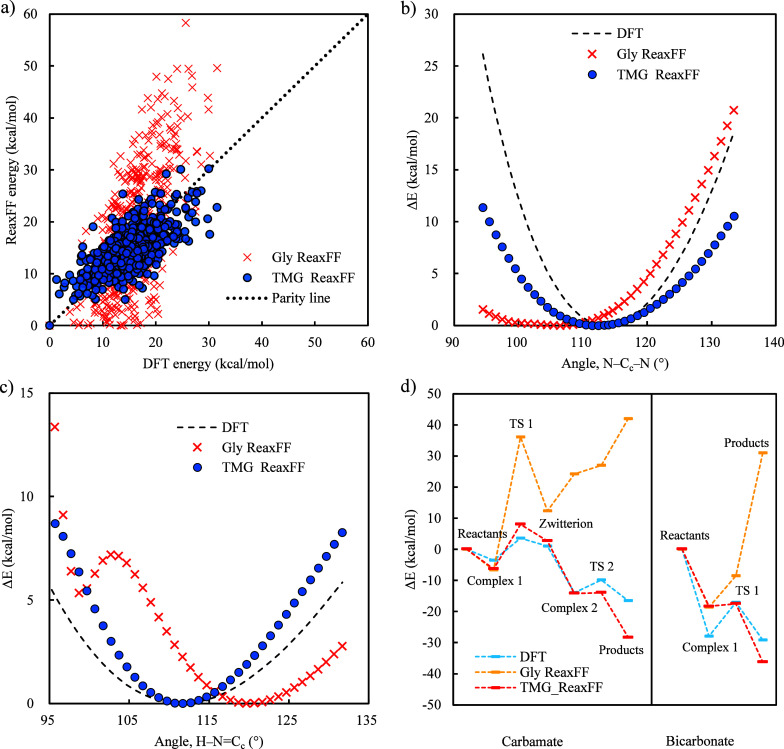
(a) Comparison of the intramolecular interactions computed by DFT, the TMG ReaxFF, and the Gly ReaxFF for a single TMG molecule. (b–c) Comparison of angle ((b) N–C_c_–N and (c) H–N=C_c_) energies calculated by DFT and those by the Gly ReaxFF and the TMG ReaxFF. The center C atom of the TMG is donated as C_c_. (d) Energy profiles along CO_2_–TMG reaction pathways calculated by DFT, the Gly ReaxFF, and the TMG ReaxFF.

Figure 5(d) also clearly shows that the iteratively optimized TMG ReaxFF can more accurately reproduce the DFT-determined energies along both reaction pathways as compared to the Gly ReaxFF (i.e., MAE values of 4.5 and 28.7 kcal/mol, respectively). Moreover, the TMG ReaxFF can describe the energy barriers and trends along both pathways, aligning with the DFT predictions. For instance, the TMG ReaxFF captures the unique preference of the reaction between CO_2_ and TMG toward the bicarbonate pathway. It is interesting to note that the best available ReaxFF in the literature—the Gly ReaxFF—even incorrectly predicts the CO_2_–TMG reaction to be unfavorable along both pathways. This again suggests the system-specific nature of ReaxFFs; their application to a new system should be carefully evaluated.

To summarize, the newly reparameterized TMG ReaxFF demonstrates excellent agreement with both experimental and DFT benchmarks. It reproduces the bulk phase density of TMG within 0.03 g/cm^3^ of the experimental value, yields a MAE of 2.9 kcal/mol across 4000 intramolecular configurations, and accurately captures reaction energy profiles along both carbamate and bicarbonate pathways (MAE = 4.6 kcal/mol).

Thus far, while the TMG ReaxFF has demonstrated strong performance in capturing the physical characteristics of TMG, as well as its reaction with CO_2_, it remains important to validate its capability in describing reactive diffusion of CO_2_ in CO_2_/TMG/H_2_O mixtures. ReaxFF MD simulations are therefore conducted to investigate the CO_2_/TMG/H_2_O systems at a water uptake value of 50 wt.% with CO_2_ loadings at 1.0, 2.5, 4.0, 5.0, and 6.0 wt.%. (denoted as simulations a–e, respectively, hereafter). In agreement with DFT calculations, the formation of zwitterion is indeed rarely observed across all simulations, and the majority of reactions follow the bicarbonate pathway. For the former, while some formation of Complex 1 and TS 1 along the carbamate pathway is indeed noted, they mostly decompose back to CO_2_ and TMG within the next few picoseconds. This implies that the formation of carbamate is difficult to achieve in TMG, indicating that carbamate formation is merely transient and inconsequential to the overall reactive diffusion. This observation is consistent with the relatively high energy barrier for the reaction between Complex 1 and TS 1 in the carbamate pathways. For the latter, interestingly, reverse reactions (e.g., from bicarbonate to CO_2_) are also observed, demonstrating that both the forward and reverse reactions can be captured. Figure 6(a) summarizes the conversion ratio of reacted CO_2_ (e.g., bicarbonate) and protonated TMG (TMG–H^+^) calculated from the last 1 ns of these simulations. The conversion ratio of reacted CO_2_ increases from ∼17% to 40% as the loading of CO_2_ increases from 1.0 wt.% to 6.0 wt.%. This is as expected, given a higher CO_2_ loading promotes the formation of bicarbonate ions in this reversible reaction. Figure 6(a) also shows an approximately 60% conversion ratio for TMG to form TMG–H^+^ through TMG–H_2_O reactions for all simulations. The computed radial density function (RDF) between the conjugated N and O (i.e., OH^–^ and H_2_O) shown in Figure 6(b) further reveals that the conjugated N is surrounded by a high concentration of OH^–^ at a distance of ∼ 2 Å (i.e., first peak). It is then followed by a high concentration of H_2_O (i.e., second peak). These indicate that the protonated TMG (TMG–H^+^) is coupled with OH^–^ and H_2_O after the TMG–H_2_O reaction. It is essential to note that under the simulation conditions, TMG molecules are not fully protonated, despite TMG is a strong base with a p*K*_a_ of ∼ 13.^41^ This partial protonation may result from a moderately hydrated environment (i.e., 50 wt.% water uptake). In such conditions, limited water availability and a decreased dielectric constant restrict the level of protonation when compared to bulk aqueous scenarios. Similar partial protonation behaviors have in fact also been observed in experimental studies of guanidine-based CO_2_ capture systems, where the balance between unprotonated and protonated species is affected by CO_2_ loading and the solvent environment.^28,49,50^

**Figure 6:**
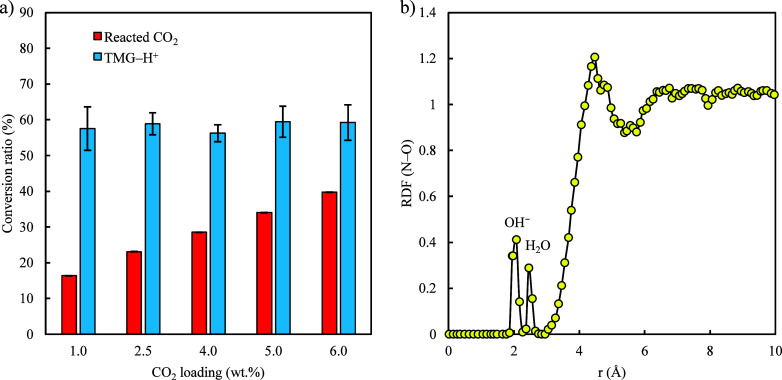
(a) The conversion ratio of reacted CO_2_ and protonated TMG at various loadings of CO_2_. (b) The RDF between the conjugated N in TMG and O (i.e., OH^–^ and H_2_O).

Unlike some other computational studies that either investigate reacted and unreacted CO_2_ separately in carriers or assume equilibrium conditions to fix the bicarbonate concentration (despite potential uncertainties in equilibrium constants),^2,14,19^ the MD simulation using the TMG ReaxFF does not require a priori assumption about the ratio of unreacted CO_2_ to bicarbonate. Instead, the reaction dynamics naturally emerge from the simulation. Furthermore, the initial CO_2_ loading in these simulations holds physical significance, as it could be connected to the gas phase CO_2_ activity, offering a more realistic depiction of CO_2_ sorption under various conditions.

### Diffusion Coefficients of CO_2_ and TMG

From the above-mentioned simulations on the CO_2_/TMG/H_2_O systems, the diffusion coefficients (*D*) of CO_2_ and TMG are calculated from the corresponding MSD with results summarized in Table 2. It should be noted that, since CO_2_ may diffuse in both unreacted and reacted (i.e., bicarbonate) forms, the diffusion coefficient of CO_2_ is calculated per the MSD of the C atom of CO_2_ regardless of its forms and listed in Table 2. Interestingly, CO_2_ is found to diffuse faster than TMG at all CO_2_ loadings; the diffusion coefficient can be as high as 10.71 × 10^–6^ cm^2^/s, which is almost twice higher than the TMG/TMG–H^+^ (i.e., an average of 5.08 × 10^–6^ cm^2^/s). Table 2 also shows that the diffusion coefficient of CO_2_ decreases as the loading of CO_2_ increases. Since the reacted CO_2_ ratio increases at higher loadings of CO_2_, it implies that the decreased diffusivity could be attributed to the more pronounced formation of bicarbonate, which may have lower mobility for its strong Coulombic interaction with TMG–H^+^.

**Table 2:** Diffusion Coefficients of CO_2_ and TMG at Various Loadings of CO_2_ and Water Uptake of 50 wt.%

		*D* (10^–6^ cm^2^/s)		
Simulation	CO_2_ loading (wt %)	CO_2_ ^a^	TMG/TMG–H^+^	Reacted CO_2_ Ratio (%)
a	1.0	10.71	5.24	16.67
b	2.5	8.46	5.07	23.33
c	3.0	8.25	5.56	28.67
d	4.0	6.99	4.86	34.17
e	6.0	6.66	4.67	40.00

a Average diffusivity of CO_2_ and HCO_3_^–^.

It is interesting to note that the calculated reactive diffusion coefficients of CO_2_ are lower than the ones measured by Mandal et al.^51^ (i.e., 7.9–19.1 × 10^–6^ cm^2^/s) in the aqueous monoethanolamine (MEA)/2-amino-2-methyl-1-propanol (AMP) solutions and MEA/*N*-methyldiethanolamine (MDEA) solutions. The TMG/TMG–H^+^ also shows a lower diffusion coefficient compared to ethylenediamine solution, measured at 8.5 × 10^–6^ cm^2^/s experimentally.^52^ The aqueous amine solutions employed in carbon capture usually exhibit lower viscosities compared to the system in this study; therefore, lower diffusivity values are expected in our moderately hydrated, high-concentration TMG systems.

To further differentiate the diffusion coefficients of unreacted CO_2_ and reacted CO_2_ (i.e., bicarbonate), additional simulations and analyses are conducted. Specifically, probing the former is challenging in our system, as sufficiently long and continuous trajectories of unreacted CO_2_ cannot be identified for a proper diffusivity determination. To overcome this, the TMG ReaxFF is modified to enhance the strength of C=O bond in CO_2_ by 20%, effectively suppressing its reactivity (hereafter referred to as inert CO_2_). It is noted that the modified TMG ReaxFF still allows the reactions between TMG and water, as well as maintains a proper description of intra/intermolecular interactions of TMG. By applying the modified TMG ReaxFF, the diffusion coefficient of inert CO_2_ is accordingly computed with the same settings as those of previous simulations. As shown in Table 3, the mobility of inert CO_2_ (i.e., 15.68 × 10^–6^ cm^2^/s) is two times higher than the reactive ones (e.g., average *D* of simulations a–e, 8.21 × 10^–6^ cm^2^/s). This supports the above interpretation that the reacted CO_2_ in the form of bicarbonate has a lower mobility.

**Table 3:** Diffusion Coefficients of Reactive CO_2_ (i.e., Average Diffusivity of CO_2_ and HCO_3_^–^), Inert CO_2_, TMG/TMG–H^+^, and Bicarbonate with and without Hopping at a Water Uptake of 50 wt.%. MSD Profiles can be Seen in SI Figure S4

Species	*D* (10^–6^ cm^2^/s)
Reactive CO_2_	8.21
Inert CO_2_	15.68
TMG/TMG–H^+^	5.08
Bicarbonate	6.53
Bicarbonate w/o hopping	4.83

For reacted CO_2_, the MSD of bicarbonate is directly calculated by selectively sampling snapshots in which bicarbonate exists. Notably, the diffusion coefficient of bicarbonate is quantified to be 6.53 × 10^–6^ cm^2^/s, 2.5 times lower than the inert CO_2_. Its mobility more closely resembles that of TMG/TMG–H^+^ with an average *D* value of 5.08 × 10^–6^ cm^2^/s. The Coulombic interactions between the bicarbonate and TMG–H^+^ indeed result in a coupling behavior that limits the mobility of bicarbonate. This result implies that the intrinsic mobility of mobile carriers (i.e., TMG in this case) is key to the design of an FTM system. Interestingly, despite the coupling effect, bicarbonate is found to still diffuse 1.5 times faster than TMG/TMG–H^+^. This prompts further investigation on the diffusion mechanism of bicarbonate, as will be detailed next.

### Diffusion Mechanism of Bicarbonate

To unravel the diffusion mechanism of bicarbonate, the distance between the C atoms of all formed bicarbonate molecules and the conjugated N atoms of neighboring TMG molecules are visualized and analyzed. Figure 7(a) depicts the diffusion trajectory of a selected bicarbonate along with its surrounding TMG/TMG–H^+^ within an 8 Å cutoff. The bicarbonate, represented by white dots, initially diffuses alongside a TMG–H^+^ molecule that is labeled in pink, demonstrating a period of coupled diffusion. Interestingly, after a certain period of time, the bicarbonate hops to a neighbor TMG–H^+^ labeled in orange and continues diffusing together with it until it hops again to another proximate TMG–H^+^ (i.e., labeled in dark red). Such distinct hopping motion essentially offers an alternative diffusion pathway for enhanced diffusivity. To quantitatively assess the contribution of these hopping events to the observed enhanced diffusivity, additional simulations are conducted by holding the relative distance between bicarbonate and its closest TMG–H^+^ a constant (i.e., resulting in a scenario where only coupled diffusion can occur). The calculated diffusion coefficient without hopping events is found to be 4.83 × 10^–6^ cm^2^/s (Table 3), which resembles, as expected per the coupling mechanism, that of TMG/TMG–H^+^ (i.e., 4.67–5.56 × 10^–6^ cm^2^/s).

**Figure 7:**
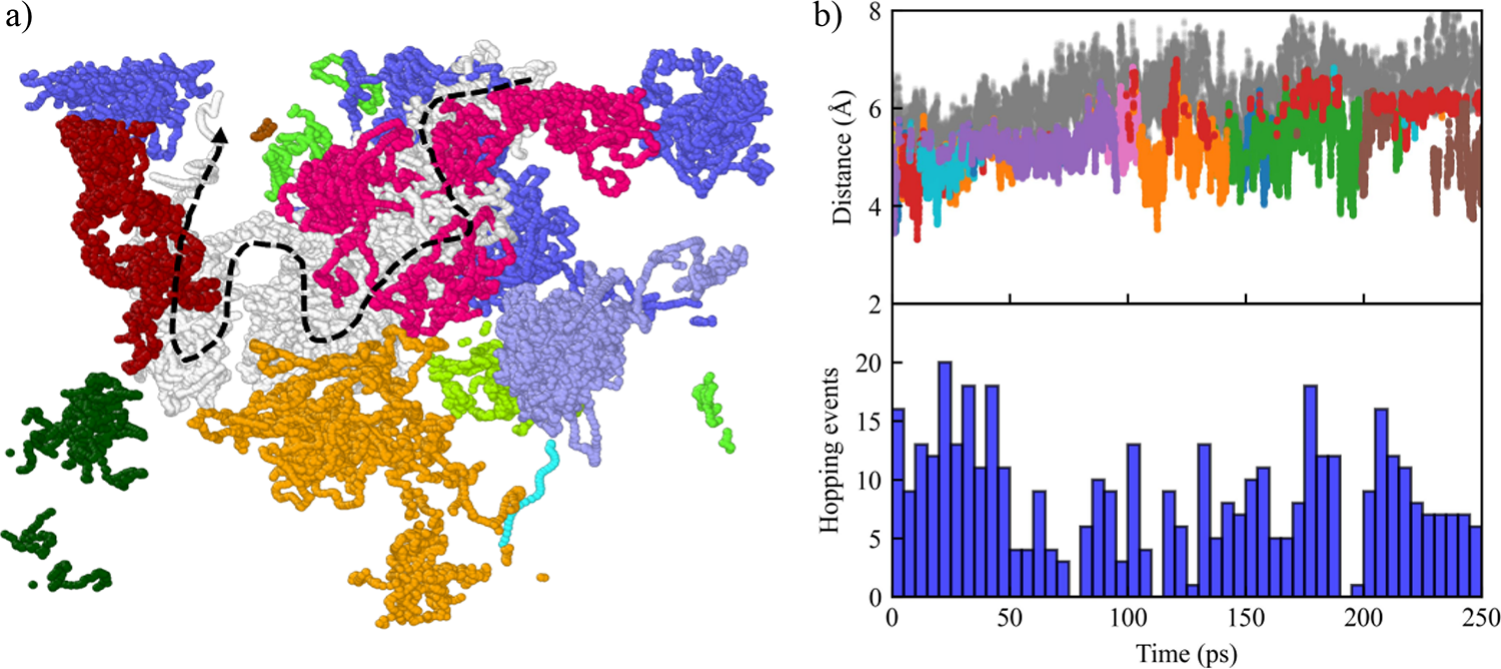
(a) Visualization of the molecular trajectory for a selected bicarbonate. As a guide to the eyes, the dashed arrow indicates the diffusion direction. Color code: white – C of the selected bicarbonate and other colors – conjugated N of TMG. Different colors for the latter represent different TMG molecules. (b) Distance between the C atom of bicarbonate and the conjugated N within its neighbors. The closest conjugated N atoms are colored in varying colors to represent different TMG molecules. The conjugated N atoms of non-nearest TMG are colored in gray for clarity. The occurrence of the hopping event is shown as a histogram with a bin size of 5 ps.

Interestingly, from tracing the nearest conjugated N atoms, Figure 7(b) shows that hopping events can occur within a very short time, which is found to be in the order of picoseconds. A hopping event is defined herein when a bicarbonate shifts to a new nearest conjugated N and does not revert to the previously paired conjugated N within 0.1 ps. Per such criteria, the hopping frequency can reach as high as 5 times per picosecond. Our results also show that the hopping events are not uniformly distributed over time, and their frequency is higher when the bicarbonate is surrounded by a higher number of different conjugated N (i.e., conjugated N in different colors in Figure 7(a)), which offers more receiving sites for the hopping events to occur. These highly frequent hopping events, which are not driven by a concentration gradient, essentially occur on a time scale comparable to the reactions, suggesting that local species dynamics should be incorporated into CO_2_ transport models. The conventional equilibrium assumption,^2,12,15–17,20^ where reaction equilibrium is expected due to slow diffusion, may fail to accurately capture the transient nature of carrier-mediated diffusion in these systems. Nonetheless, the results in this study suggest that CO_2_ diffusion in FTMs is characterized by distinct, rapid hops between carrier sites, potentially leading to deviation in concentration distribution from those predicted by macroscopic continuum mechanics. This finding not only explains why the diffusion coefficient of bicarbonate is greater than that of TMG but also paves the way for the future design of FTM systems with enhanced performance.

## Conclusion

This work represents one of the first molecular investigations into the reactive diffusion of CO_2_ in mobile carriers. Considering the weaker interspecies interactions of bicarbonates compared to carbamates, this study specifically examines their diffusion behavior in the presence of a guanidine-based mobile carrier TMG. State-of-the-art MD simulations are conducted, and these calculations employ a bond-order-based reactive force field with parameters reparameterized against references from experimental density and density functional theory calculations. The force field, denoted as the TMG ReaxFF achieved in this study, can reasonably describe inter/intramolecular interactions of TMG molecules, energy profiles of key species along the reaction pathways with CO_2_, as well as reaction preferences in CO_2_/TMG/H_2_O mixtures. The simulation results show that bicarbonate ions diffuse along with protonated TMG in their proximity, following the conventionally anticipated coupling mechanism. As such, the diffusion of reacted CO_2_ is highly subject to the intrinsic mobility of carriers. Importantly, while bicarbonate ions are strongly coupled with protonated TMG, they can also hop between protonated carriers. This effectively offers an alternative pathway for diffusion, and the occurrence of such hopping events is found to enhance the diffusion coefficient of reacted CO_2_ by as much as 40%. Overall, these findings provide direct insight into the diffusion mechanisms of bicarbonate in FTMs, offering guidance for the design of mobile carriers to enhance CO_2_ separation efficiency.

## Supplementary Material





## Notes

Molecular structures of TMG, performance of ReaxFFs, MSD profiles, details of water solvation models (PDF)
Force field parameters of the TMG ReaxFF (TXT)
