# Supplementary material for: Atomistic Insights into the Reactive Diffusion of CO2 in Guanidine-Based Facilitated Transport Membranes
Source: J Phys Chem C Nanomater Interfaces. 2025 May 11;129(20):9550–61. doi: 10.1021/acs.jpcc.5c01717 (PMC12105039; doi:10.1021/acs.jpcc.5c01717)
Supplement: Supplementary file 1 [file jp5c01717_si_001.pdf]

Supporting Information

for

Atomistic Insights into the Reactive Diffusion of CO_2 in Guanidine-Based Facilitated Transport Membranes

Changlong Zou[a], Xuepeng Deng[a], Yang Han[a,], Li-Chiang Lin[a,b,*]*

[a] *William G. Lowrie Department of Chemical and Biomolecular Engineering, The Ohio State University, 151 West Woodruff Avenue, Columbus, OH 43210-1350, USA.*

[b] *Department of Chemical Engineering, National Taiwan University, No.1, Sec. 4 Roosevelt Rd. Taipei 10617, Taiwan.*

*Email: Y. Han (han.779@osu.edu); L.-C. Lin (lclin@ntu.edu.tw)

Contents

1. Figures referred to in the main text..S3

2. Water solvation models...S7

3. References...S10

1. Figures referred to in the main text

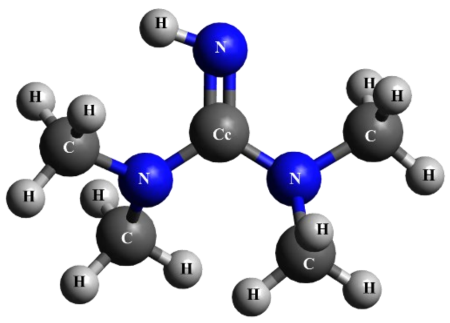

Figure S1. Molecular structure of 1,1,3,3-tetramethylguanidine (TMG). The center, conjugated carbon is labelled as C_c.

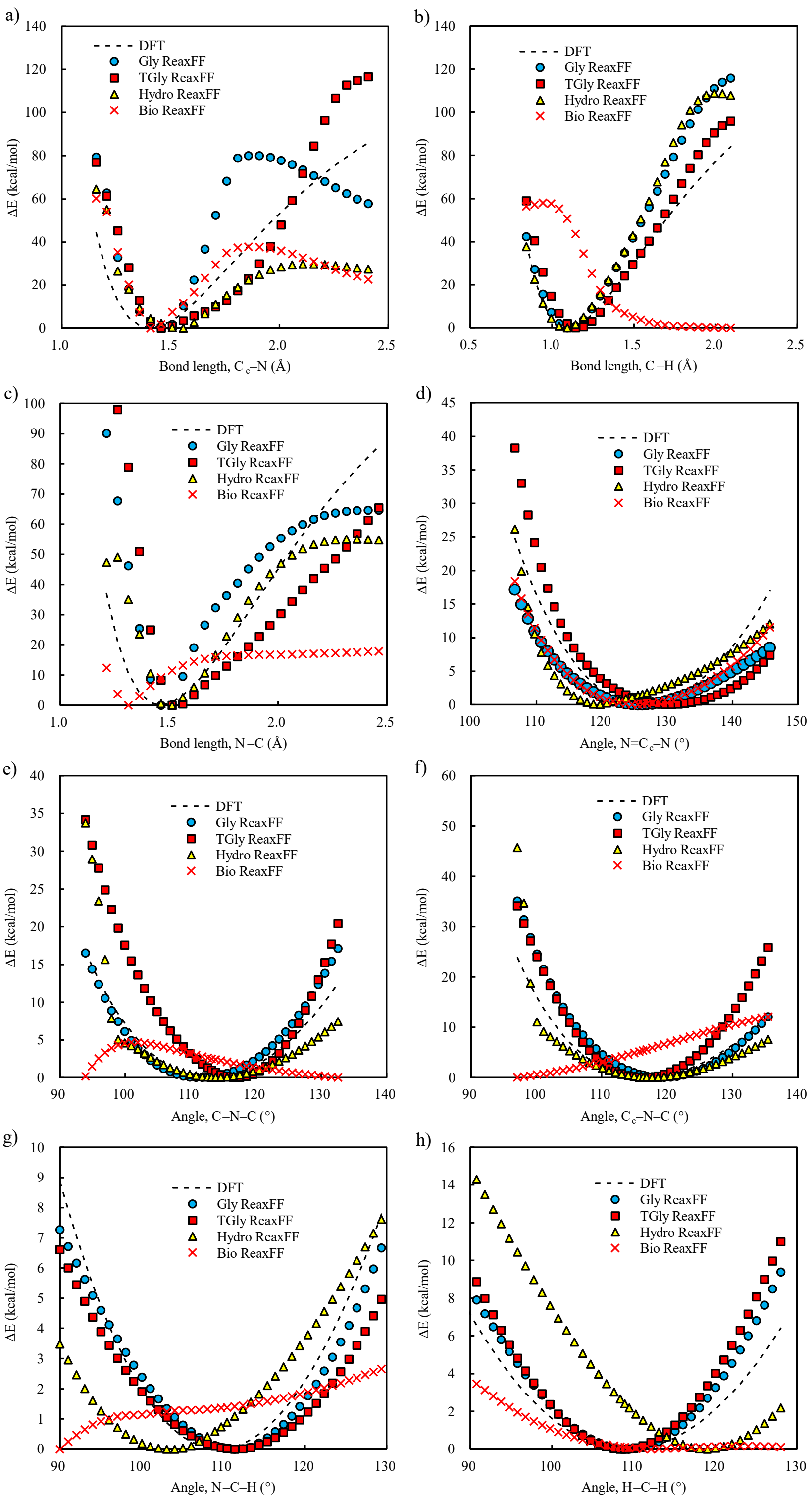

Figure S2. DFT- and ReaxFF-computed energy as a function of selected bonds and angles for a single TMG molecule. ReaxFFs considered in this comparison include the Gly ReaxFF[1], the TGly ReaxFF[2], the Hydro ReaxFF[3], and the Bio ReaxFF[4].

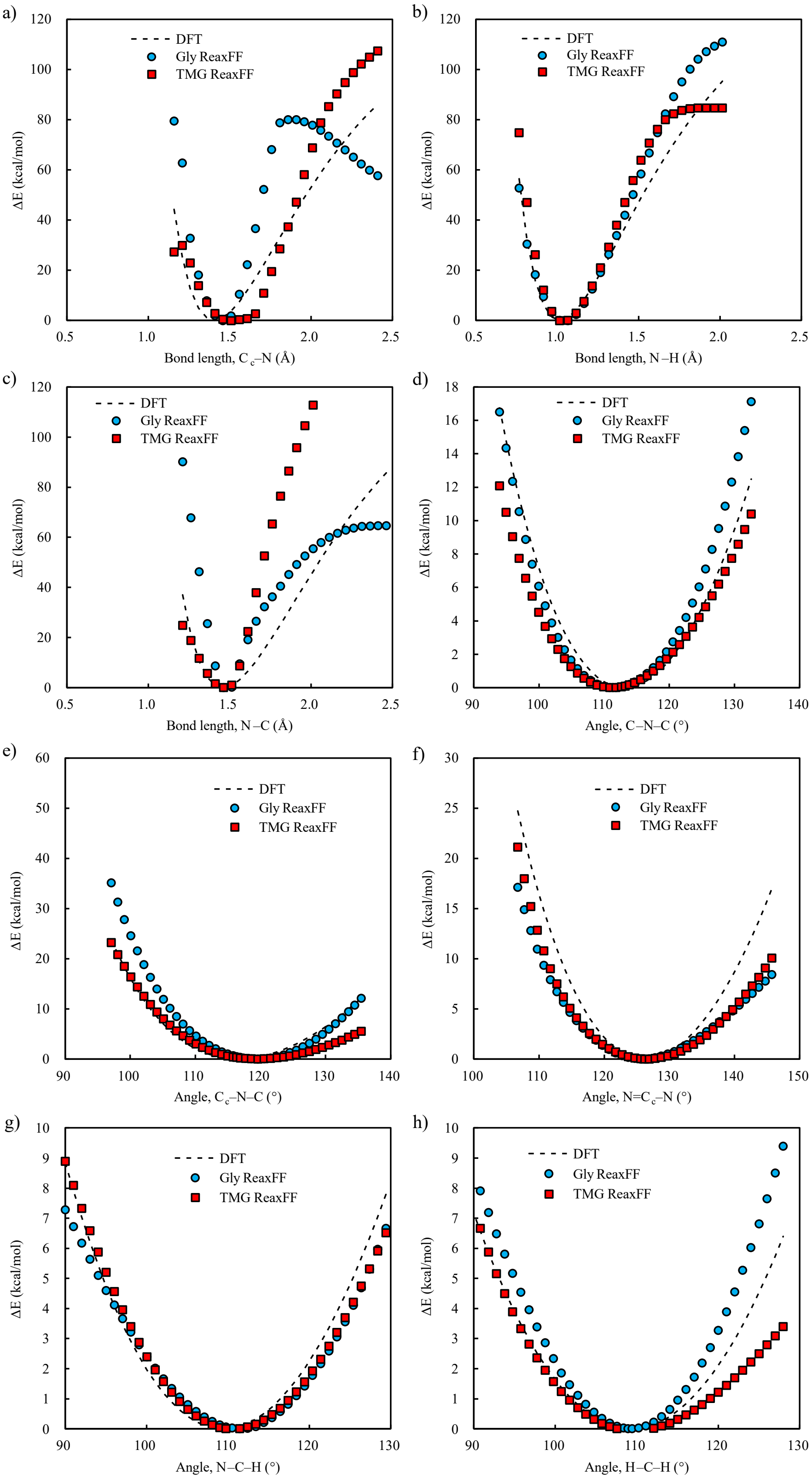

Figure S3. DFT- and ReaxFF-computed energy as a function of selected bonds and angles for a single TMG molecule. ReaxFFs considered in this comparison include the Gly ReaxFF[1] and the newly parametrized TMG ReaxFF.

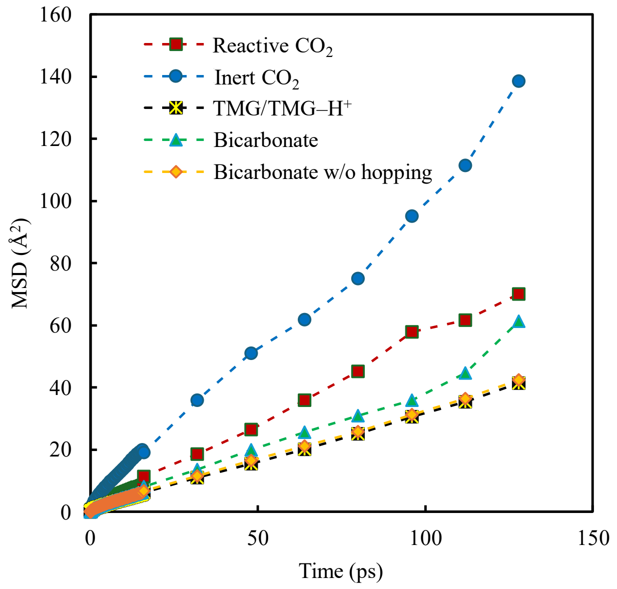

Figure S4. Mean squared displacement (MSD) of reactive CO_2, inert CO_2, TMG/TMG–H^+, and bicarbonate (with or without hopping) as a function of time from selected MD simulations. The time length is chosen based on the period when bicarbonate exists.

2. Water solvation models

Water plays an important role in the CO_2-carrier reactions since it is not only involved in the bicarbonate pathway as a reactant but also stabilizes the intermediates and products. In DFT calculations, water solvation is generally modelled explicitly or implicitly. In the explicit solvation approach, the solvation shell is constructed with a large number of individual water molecules. Therefore, it can capture the local solvation features (e.g., hydrogen bonds). However, the explicit solvation model may be prohibitively expensive and inefficient, particularly when a large number of explicit water molecules are considered. On the other hand, the implicit solvation model can greatly reduce the computational complexity by describing the solvation shell as a continuous medium. This approach has been parameterized to perform similarly to that of the explicit solvation model and is widely adopted.[5,6] However, its reliability in capturing hydrogen bonds could still be a concern.[7]

To this end, a hybrid approach, which introduces a small number of explicit water molecules in an implicit solvation model, has been employed herein. Specifically, explicit water molecules are strategically placed around hydrogen bond donors and acceptors (e.g., $–COO^-$ of the carbamate product and the bicarbonate ion of the bicarbonate product), followed by structural relaxation using DFT. As explicit water molecules develop hydrogen bonds with the solute molecules, the hybrid solvation model (i.e., N>0), as expected, yields stronger solvation energies as compared to the implicit solvation model (i.e., N=0). As shown in Figure S5 the hybrid solvation model, especially when N>4, shows similar product stabilities following the carbamate and bicarbonate pathways (i.e., $\Delta E_{carbamate}$ and $\Delta E_{bicarboante}$, respectively) and reaction preference (i.e., $\Delta\Delta E = E_{carbamate} - \Delta E_{Bicarboante}$). In this study, eight explicit water molecules are employed in the calculations of CO_2–TMG reaction pathways.

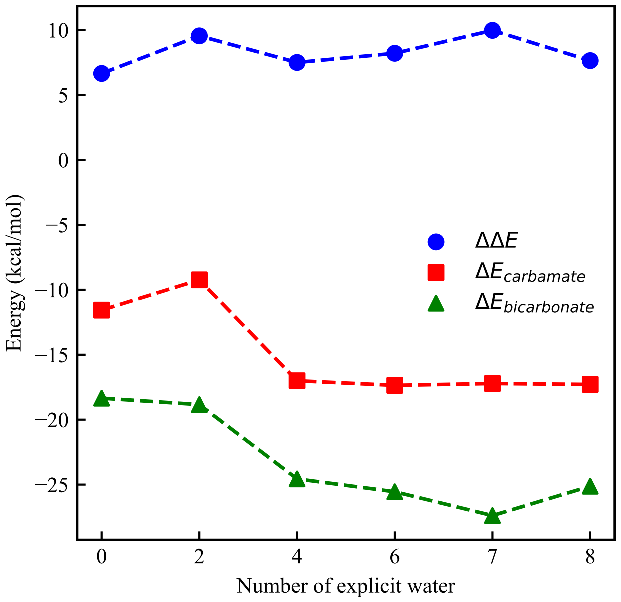

Figure S5. The preference of TMG–CO_2 reaction pathways determined using DFT with different numbers of explicit water molecules (N). Note that when N=0, a purely implicit model is adopted. In this figure, $\Delta E_{carbamate}$ and $\Delta E_{bicarbonate}$ represent the energy difference between the product and reactant along the carbamate and bicarbonate pathway, respectively. A lower value means a more thermodynamically favorable reaction. ΔΔE represents the reaction preference, i.e., $\Delta E_{carbamate} - \Delta E_{bicarbonate}$.

The performance of ReaxFFs in describing the DFT-computed solvation energies is also evaluated with results summarized in Figure S6 The TMG ReaxFF has a decent MAE of 10.7 kcal/mol, notably better than that of the Gly ReaxFF (i.e., 17.3 kcal/mol).

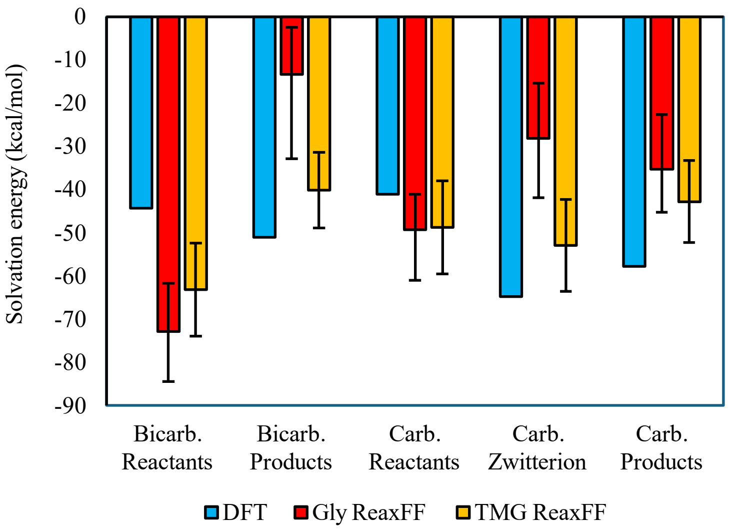

Figure S6. Comparison of the water solvation energy calculated by DFT with a hybrid solvation model against those calculated by the Gly ReaxFF[2] and the TMG ReaxFF developed in this study.

3. References

(1) Rahaman, O.; van Duin, A. C. T.; Goddard, W. A.; Doren, D. J. Development of a ReaxFF Reactive Force Field for Glycine and Application to Solvent Effect and Tautomerization. *J. Phys. Chem. B* 2011, *115* (2), 249–261.

(2) Zhang, W.; van Duin, A. C. T. Improvement of the ReaxFF Description for Functionalized Hydrocarbon/Water Weak Interactions in the Condensed Phase. *J. Phys. Chem. B* 2018, *122* (14), 4083–4092.

(3) Zhang, Z.; Rao, S.; Han, Y.; Pang, R.; Ho, W. S. W. CO2-Selective Membranes Containing Amino Acid Salts for CO2/N2 Separation. *J. Membr. Sci.* 2021, *638*, 119696.

(4) Carrera, G. V. S. M.; Jordão, N.; Branco, L. C.; da Ponte, M. N. CO2 Capture and Reversible Release Using Mono-Saccharides and an Organic Superbase. *J. Supercrit. Fluids* 2015, *105*, 151–157.

(5) Lei, X.; Xu, Y.; Zhu, L.; Wang, X. Highly Efficient and Reversible CO2 Capture through 1,1,3,3-Tetramethylguanidinium Imidazole Ionic Liquid. *RSC Adv.* 2014, *4* (14), 7052–7057.

(6) Lee, Y. Y.; Wickramasinghe, N. P.; Dikki, R.; Jan, D. L.; Gurkan, B. Facilitated Transport Membrane with Functionalized Ionic Liquid Carriers for CO2/N2, CO2/O2, and CO2/Air Separations. *Nanoscale* 2022, 14 (35), 12638–12650.

(7) Dasgupta, N.; Yilmaz, D. E.; van Duin, A. Simulations of the Biodegradation of Citrate-Based Polymers for Artificial Scaffolds Using Accelerated Reactive Molecular Dynamics. *J. Phys. Chem.* B 2020, 124 (25), 5311–5322.

(8) Davran-Candan, T. DFT Modeling of CO2 Interaction with Various Aqueous Amine Structures. *J. Phys. Chem.* A 2014, 118 (25), 4582–4590. Klemm, A.; Lee, Y. Y.; Mao, H.; Gurkan, B. Facilitated Transport Membranes with Ionic Liquids for CO2 Separations. *Front. Chem.* 2020, 8, 637.

(9) Narimani, M.; Amjad-Iranagh, S.; Modarress, H. CO2 Absorption into Aqueous Solutions of Monoethanolamine, Piperazine, and Their Blends: Quantum Mechanics and Molecular Dynamics Studies. *J. Mol. Liq.* 2017, 233, 173–183.

(10) Da Silva, E. F.; Svendsen, H. F.; Merz, K. M. Explicitly Representing the Solvation Shell in Continuum Solvent Calculations. *J. Phys. Chem. A* 2009, 113 (22), 6404–6409.
